# Urinary Exosomal MicroRNA Profiling in Incipient Type 2 Diabetic Kidney Disease

**DOI:** 10.1155/2017/6978984

**Published:** 2017-09-05

**Authors:** Yijun Xie, Yijie Jia, Xie Cuihua, Fang Hu, Meng Xue, Yaoming Xue

**Affiliations:** Department of Endocrinology and Metabolism, Nanfang Hospital, Southern Medical University, Guangzhou, Guangdong 510515, China

## Abstract

**Background:**

Albuminuria is an early sign but not a strong predictor of diabetic kidney disease (DKD). Owing to their high stability, urinary exosomal miRNAs can be useful predictors of the progression of early-stage DKD to renal failure; fluid biopsies are ideal for detecting abnormalities in these miRNAs. The aim of this study was to identify novel differentially expressed miRNAs as urine biomarkers for type 2 DKD by comparing between patients of type 2 diabetes (T2D) with and without macroalbuminuria.

**Methods:**

Ten patients with T2D, including five who had no renal disease and five with macroalbuminuria (DKD G1-2A3), were selected for this study. Exosome- (UExo-) derived miRNA profiles were used to identify candidate biomarkers, a subset of which was verified using quantitative reverse transcription PCR.

**Results:**

A total of 496 UExo-derived miRNA species were found to be differentially expressed (>2-fold) in patients with DKD, compared to those with T2D. A validation analysis revealed that three miRNAs (miR-362-3p, miR-877-3p, and miR-150-5p) were upregulated and one (miR-15a-5p) was downregulated. These miRNAs might regulate DKD through p53, mTOR, and AMPK pathways.

**Conclusions:**

In conclusion, UExo-derived miRNAs were altered in type 2 DKD. MiR-362-3p, miR-877-3p, miR-150-5p, and miR-15a-5p might be novel biomarkers for incipient DKD.

## 1. Introduction

Diabetic kidney disease (DKD) is a type of chronic kidney disease (CKD) caused by diabetes mellitus. It is the leading cause of end-stage renal diseases in Western countries [1] and has been reported in more than 30% of patients with type 2 diabetes mellitus (T2DM) in China [2]. DKD has an insidious onset; once proteinuria occurs, progression to end-stage renal disease is rapid. Microalbuminuria does not accurately predict DKD; [3] new biomarkers to identify the early stage of DKD are therefore urgently needed.

MicroRNAs (miRNAs) are a group of short (~22 nt), small, noncoding RNAs that posttranscriptionally regulate gene expression by suppressing target mRNAs [4]. Previous experimental studies have suggested the involvement of miRNAs with the pathogenesis of renal diseases [5, 6] and the development of DKD [7]. Cell-free circulating miRNAs are known to be stable in a variety of body fluids, including urine. Urine is a suitable source of biomarkers for kidney diseases, and several urinary miRNA biomarkers have been identified for IgA nephropathy [8], nephrotic syndrome [9], lupus nephritis [10], and DKD in type 1 diabetes mellitus [11].

Exosomes (40–100 nm) are cup-shaped vesicles derived from the cellular endocytic compartment that can be isolated from urine and other body fluids such as serum, plasma, saliva, and milk [12]. Because exosomes can carry proteins, nucleotides, deoxynucleotides, and miRNAs to distant target cells, they represent an important mechanism for cell-to-cell communication [13]. Urinary exosome- (UExo-) derived miRNAs may be better diagnostic markers than free miRNAs. UExo-derived miRNAs are protected from endogenous RNase activity, are remarkably stable, and are not easily confounded by plasma miRNAs that pass the glomerular filtration barrier [14]. Changes in UExo-derived miRNAs have been found to be significantly correlated with the progression of focal segmental glomerulosclerosis [15] and DKD in type 1 diabetes mellitus [16].

In vitro studies and analyses of the urinary exosomes of patients with T2DM have shown that increased levels of miR-192 and miR-215 promote renal injury in DKD [17]. It is to be noted that neither free urinary miRNA profiling in patients with type I DKD [18] nor UExo-derived miRNA profiling in patients with T1 [16] and T2 [19] DKD has been able to verify the association between miR-192 and DKD.

Our previous studies [20] have shown that the expression of UExo-derived miR-192 increased in patients with T2DM with microalbuminuria but decreased in those with macroalbuminuria. The combined analysis of the expression levels of UExo-derived miR-192 and TGF-*β*1 might provide new insights into the pathology of incipient DKD. The underlying mechanism of the association between UExo-derived miRNAs and DKD pathogenesis deserves exploration.

Circulating levels of total and specific miRNAs are known to be lower in patients with severe chronic renal failure than in those with mild renal impairment or normal renal function [14]. We therefore assumed that the initially upregulated UExo-derived miRNAs in patients with renal failure would be downregulated.

It is important to consider kidney function when identifying miRNAs as biomarkers of DKD. Previous analyses of the miRNA signature in urinary exosomes of patients with type 2 DKD [19] have focused on the decline in glomerular filtration rate (GFR). Differences in UExo-derived miRNAs between patients with normal or mildly decreased eGFR (G1 or G2) and those with severely increased albuminuria (A3) [21] are unclear. Here, we aimed to identify novel miRNA candidates that reflected changes in incipient type 2 DKD; these miRNAs could have implications in preventing or delaying disease progression.

## 2. Materials and Methods

### 2.1. Patient Characteristics

Ten patients, who were diagnosed with T2DM and admitted to the Department of Endocrinology and Metabolism, Nanfang Hospital, Southern Medical University, Guangdong, China, from January to November 2016, were enrolled for the study. The exclusion criteria were as follows: type 1 diabetes mellitus; nondiabetic kidney disease; acute diabetic complications (diabetic ketoacidosis, hyperosmolar nonketotic coma, and inflammatory disorders); diabetes mellitus complicated with severe heart, liver, or renal insufficiency (uremia); neoplastic disorders; urinary tract infection; rheumatic diseases; use of nephrotoxic drugs; hepatitis B; pregnancy; stroke; and occlusive peripheral vascular disease. Patients with an eGFR < 60 mL/min/1.73 m^2^ and those with macroalbuminuria or microalbuminuria who did not present with diabetic retinopathy were also excluded.

Urine samples were obtained from each patient. For the miRNA array, patients were classified into two groups based on the degree of albuminuria, a normoalbuminuria group (urine albumin-to-creatinine ratio (ACR) < 2.5 mg/mmol and urinary albumin excretion rate (AER) < 30 mg/24 h, *N* = 5) and a macroalbuminuria group (ACR > 25 mg/mmol or AER = 300–800 mg/24 h, *N* = 5).

Thirty additional patients with T2DM were enrolled for verification using qRT-PCR. The exclusion criteria were the same as those described previously, but only patients with eGFR < 60 mL/min/1.73 m^2^ were selected. Thus, 40 patients were classified into two groups, a normoalbuminuria group (ACR < 2.5 mg/mmol and AER < 30 mg/24 h, *N* = 20) and a macroalbuminuria group (ACR > 25 mg/mmol or AER > 300 g/24 h, *N* = 20).

This study was approved by the ethics committee of Nanfang Hospital, Southern Medical University. All patients provided written informed consent prior to participating in the study.

### 2.2. Urinary Exosomal Isolation

Initially, 100 mL of first-morning urine was collected from all subjects in sterile containers. All the samples were processed within 1 h of collection. Urinary cells were removed by centrifugation at 2000 ×*g* (Rotor: JA-20; Beckman Coulter, Fullerton, CA, USA) for 15 min at 4°C and at 10,000 ×*g* for 15 min at 4°C. Subsequently, the supernatant was ultracentrifuged at 170,000 ×*g* for 70 min at 4°C (Rotor: SW 32Ti; Beckman Coulter, Brea, CA, USA). After removing the supernatant, the pellets were washed with 8 mL of sterile phosphate-buffered saline and ultracentrifuged at 170,000 ×*g* for 70 min. The pellets were then suspended in 100 *μ*L of phosphate-buffered saline and stored at −80°C for further analyses.

### 2.3. Total RNA Extraction

The UExo samples were treated with 0.1 *μ*g/*μ*L RNase A (BioSharp, USA) for 10 min at 37°C. Total RNA was isolated using TRIzol (Invitrogen/DingGuo, Beijing, China) and the miRNeasy Mini Kit (QIAGEN, Hilden, Germany), according to the manufacturer's instructions. The extracted RNA was quantified and assessed using a NanoDrop ND-1000 Spectrophotometer (Thermo Fisher Scientific, Wilmington, DE, USA). Quality control was performed using the Agilent 2100 Bioanalyzer.

### 2.4. miRNA Profiling and Data Analysis

After quality control, the miRCURY™ Hy3™/Hy5™ Power Labeling Kit (Exiqon, Vedbaek, Denmark) was used to make a 25 *μ*L mixture containing 1 *μ*L of RNA, according to the manufacturer's guidelines for miRNA labeling. After labeling, the Hy3-labeled samples were hybridized on the miRCURY LNA Array (v.19.0) (Exiqon). Finally, the slides were scanned using the Axon GenePix 4000B Microarray Scanner (Axon Instruments, Foster City, CA, USA). The scanned images were then imported into GenePix Pro 6.0 software (Axon) for grid alignment and data extraction. After normalization, miRNAs that showed significant differential expression between the two groups were identified based on fold changes > 2 and *P* values < 0.05. Hierarchical clustering was performed to identify differences in the miRNA expression profiles among samples.

### 2.5. Animals and Treatment

Six male C57BL/6J mice (3-4-week old, weighing 15-16 g) were obtained from the Animal Center of Guangdong province. After four weeks on high-fat diets, the mice received a single injection of STZ (120 mg/kg, i.p., in citrate buffer, pH = 4.5, MP Biomedicals). Blood glucose was measured weekly and was found to have reached a sustained level of >16.7 mM at 16 weeks, which was considered an indicator of hyperglycemia. The mice in the DM group were sacrificed after modeling (*N* = 3); twelve weeks later, the mice of the DKD group were also sacrificed (*N* = 3). Renal cortex samples were snap frozen in liquid nitrogen and stored at −80°C for analysis. The protocols for all animal studies conformed to the established institutional and state guidelines for the care and use of laboratory animals. Total RNA was obtained from renal cortex tissues using the TRIzol reagent, as described previously.

### 2.6. Quantitative Reverse Transcription PCR

The miRNAs were reverse transcribed to complementary DNA (cDNA) using the miRcute miRNA First-strand cDNA Synthesis Kit (TIANGEN, Beijing, China). RT-PCR was performed using the miRcute miRNA qPCR Detection Kit (TIANGEN) and the LightCycler480 Real-Time PCR System (Roche; Hoffmann-La Roche Ltd., Basel, Switzerland). The relative expression levels of the target miRNAs were calculated by the comparative 2^−ΔΔCT^ method with U6 snRNA as the internal control. Primers for miR-362-3p, miR-877-3p, miR-150-5p, miR-15a-5p, and U6 were obtained from TIANGEN.

### 2.7. Bioinformatics Analysis of miRNA Target Genes and Pathways

#### 2.7.1. Identification of miRNA Target Genes

To infer the putative targets of differentially expressed miRNAs, two algorithms were used: targetScan7.1 (http://www.targetscan.org/vert_71) and mirdbV5 (http://mirdb.org/miRDB). Genes for which consistent results were obtained using both databases were considered target genes of the miRNAs. An interaction network of differentially expressed miRNAs and predicted target genes was constructed using Cytoscape v2.8.3 (http://cytoscape.org).

#### 2.7.2. Functional Enrichment Analysis

To investigate the biological functions of the target genes, a Gene Ontology (GO) function and Kyoto Encyclopedia of Genes and Genomes (KEGG) pathway enrichment analyses were performed using the DAVID tool (https://david.ncifcrf.gov/). *P* < 0.05 and FDR < 0.05 was considered statistically significant.

### 2.8. Statistical Analyses

All the data were analyzed using the SPSS 20.0 software. All results are presented as mean ± SEM. The results of the qRT-PCR were analyzed using Student's *t*-tests. CT values above 45 were considered undetectable, and *P* < 0.05 was considered significant.

## 3. Results

### 3.1. Patient Characteristics

Clinical and laboratory characteristics of the participants are summarized in Table 1. No significant differences were observed between the normo- and macroalbuminuric groups in age, gender, SBP, DBP, BMI, serum creatinine, eGFR, triglycerides, cholesterol, LDL-C, and HDL-C. Although the duration of diabetes and the HbA1c values were greater in the macroalbuminuric patients than in the normoalbuminuric ones, these differences were not statistically significant. As expected, the ACR (*t* = 7.619, *P* < 0.001) and AER (*t* = 15.724, *P* < 0.001) values were higher in patients with macroalbuminuria than in those with normoalbuminuria.

### 3.2. Urinary Exosome Characteristics

**Figure 1:**
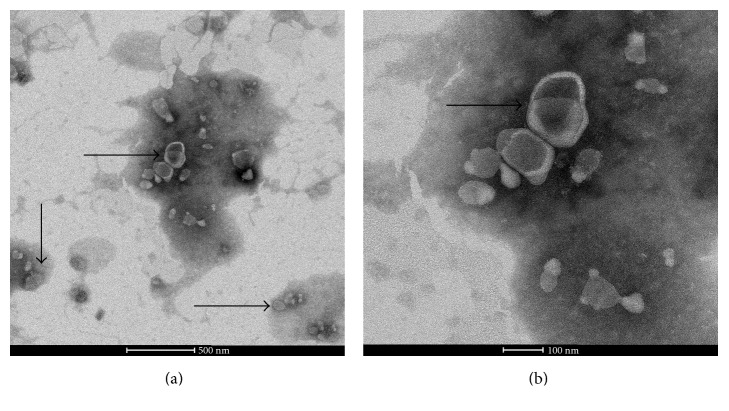
Identification of urinary exosomes by transmission electron microscopy.

Vesicles isolated from the urine collected overnight were less than 100 nm in size, with a characteristic cup-shaped morphology, as observed by electron microscopy (Figure 1). The total RNA extracted from exosomes was analyzed using an Agilent 2100 Bioanalyzer, and the urinary exosomes were found to be rich in miRNAs (Figure S1 in Supplementary Material available online at https://doi.org/10.1155/2017/6978984).

 

### 3.3. Differentially Regulated UExo-Derived miRNAs in Patients with Type 2 DKD

**Figure 2:**
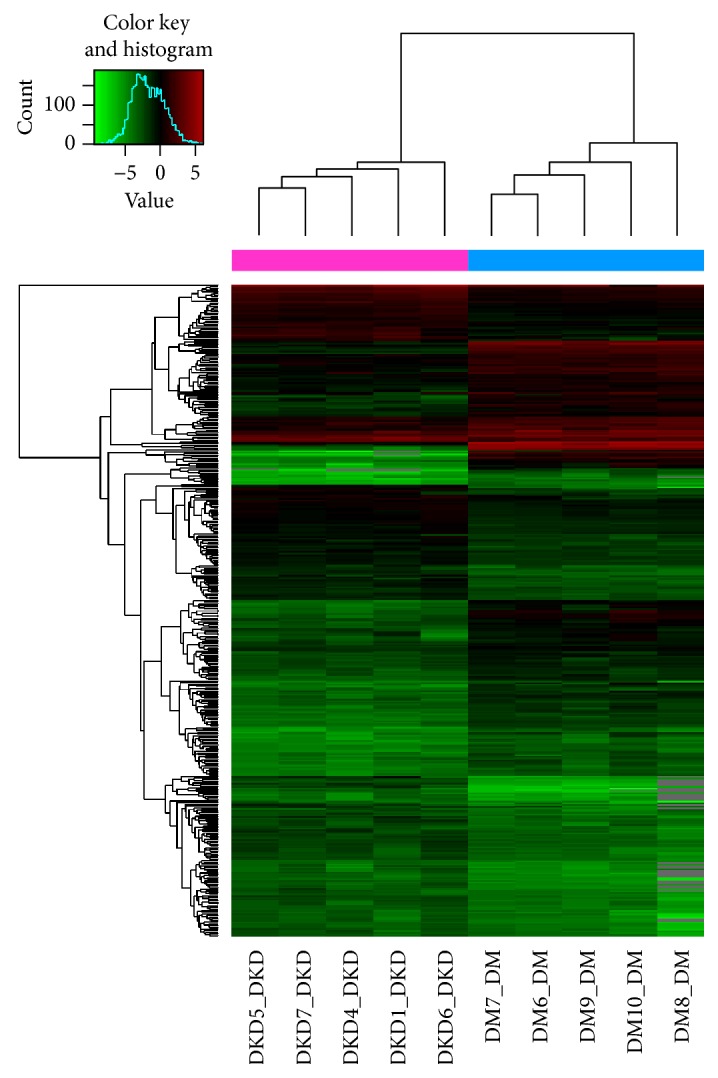
Clustering analysis of miRNA expression in urinary exosomes (DKD versus DM).

In total, 3100 miRNAs were detected in the urinary exosomes of the macro- and normoalbuminuric patients with T2DM. Differential miRNA profiling revealed 496 miRNAs that were differentially expressed at least 2-fold, in matched pairs of macro- and normoalbuminuric patients, including 203 upregulated miRNAs and 188 downregulated ones (Table 2, Figure 2). For biological analysis, we selected two miRNAs that showed the most significant differential expression, hsa-miR-362-3p and hsa-miR-3191-5p. We also selected two miRNAs that were differentially expressed in both CKD [22] and DKD, hsa-miR-877-3p and hsa-miR-15a-5p. Based on the promoter-binding region upstream of the miRNAs, six miRNAs associated with the NF-*κ*B pathway were selected, namely, hsa-miR-150-5p, hsa-miR-186-5p, hsa-miR-491-5p, hsa-miR-133b, hsa-miR-638, and hsa-miR-324-3p. MiR-362-3p is known to be upregulated in human kidney allografts with tubulointerstitial fibrosis [23]. Previous studies have shown an association between miR-150-5p and proteinuria and eGFR decline [24]. To validate the differentially expressed miRNAs, qRT-PCR was performed for miR-362-3p, miR-877-3p, miR-15a-5p, and miR-150-5p.

### 3.4. Quantitative Reverse Transcription PCR

**Figure 3:**
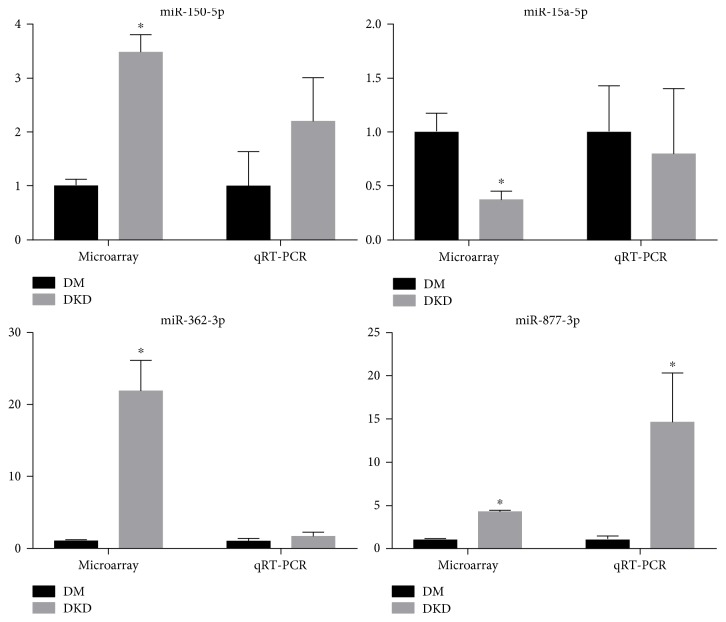
Fold changes in the expression levels of UExo-derived miRNAs. Significant differences against the DM and DKD groups are indicated by ^∗^*P* < 0.05.

**Figure 4:**
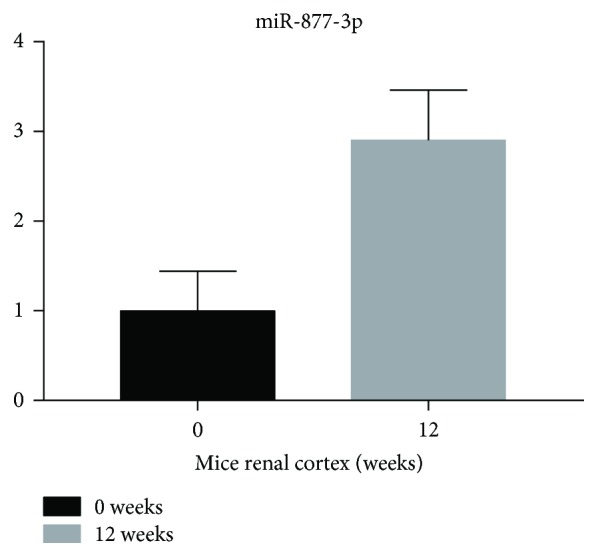
Expression levels of miR-877-3p in mice renal cortex.

A total of 20 patients with T2DM and 20 patients with DKD were recruited to confirm the results obtained for the screening cohort. The expression levels of UExo-derived miR-362-3p, miR-877-3p, miR-150-5p, and miR-15a-5p were analyzed by qRT-PCR. The expression of miR-877-3p was found to be significantly upregulated in the UExos from patients with DKD, compared to those with T2DM, whereas the expression levels of miR-362-3p, miR-150-5p, and miR-15a-5p were not significantly different in the different groups (Figure 3). We then detected the differentially expressed UExo-derived miR-877-3p in the renal cortex of diabetic mice. The mice with DKD showed higher levels of urinary microalbumin and had higher renal weight index, than those in the DM group (Hu et al. unpublished data). As expected, miR-877-3p was upregulated in diabetic kidneys, but not in a statistically significant manner (*P* = 0.056, Figure 4). Overall, the qRT-PCR and miRNA array results were consistent, indicating that the miRNA array was reliable.

### 3.5. Target Gene Detection and Functional GO and Pathway Enrichment Analyses

**Figure 5:**
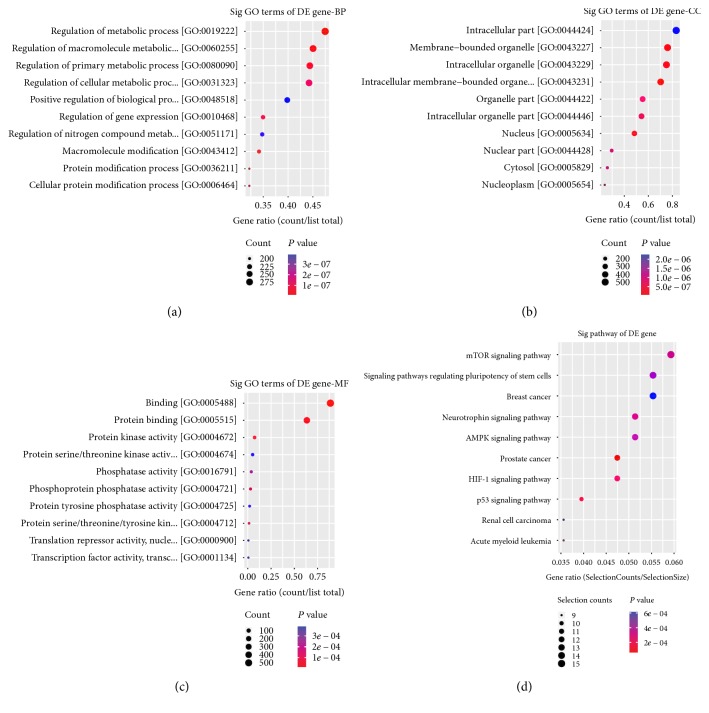
Gene ratio dot plot.

A total of 724 target genes were predicted by both targetScan7.1 and mirdbV5 for the 10 selected miRNAs. An interaction network was constructed using Cytoscape (Figure S2). We performed a GO analysis for the target genes of the 10 miRNAs. Target genes of miRNAs in three major categories, biological process (BP), cellular component (CC), and molecular function (MF), were analyzed. In the BP category, significant enrichment of target genes was detected for macromolecular modification (GO: 0019222, *P* = 3.90*e* − 09, count: 292) and regulation of metabolic processes (GO: 0019222, *P* = 3.36*e* − 09, count: 210) (Figure 5(a)). Membrane-bounded organelles (GO: 0043227, *P* = 9.55*e* − 08, count: 488) and intracellular membrane-bounded organelles (GO: 0043229, *P* = 2.21*e* − 07, count: 482) were the most relevant groups in the CC category (Figure 5(b)). Binding (GO: 0005488, *P* = 5.46523*e* − 08, count: 547) and protein binding (GO: 0005515, *P* = 2.31*e* − 06, count: 391) in the MF category were the most significantly enriched terms associated with the target genes (Figure 5(c)).

**Figure 6:**
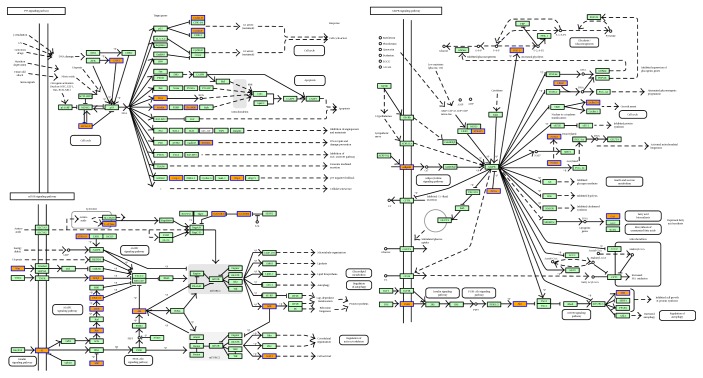
KEGG pathways of p53, mTOR, and AMPK.

We also performed a KEGG pathway enrichment analysis for differentially expressed miRNA target genes, with FDR < 0.05 as the criterion for pathway detection. The target genes showed enrichment for 54 pathways, particularly pathways associated with malignant tumors, p53, neurotrophic factors, mTOR, AMPK, and other signaling pathways. The most highly enriched pathways were the mTOR signaling pathway (FDR = 0.01717889) and the signaling pathways that regulate the pluripotency of stem cells (FDR = 0.01792762) (Figures 5(d) and 6).

## 4. Discussion

Consistent with the results obtained here, UExos do indeed contain large amounts of miRNAs. We extracted UExos using a two-step differential ultracentrifugation process, which is considered ideal for exosome isolation from body fluids [25]. The size and features of the exosomes were determined by electron microscopy. We detected differences in UExo-derived miRNA profiles between patients with T2DM with and without macroalbuminuria, including 203 upregulated miRNAs and 188 downregulated ones, suggesting that UExo-derived miRNA signatures could be used as noninvasive biomarkers for predicting and diagnosing DKD.

Previous studies have explored UExo-derived miRNA profiles in patients with T2DKD with decreased renal functions. Albuminuria is apparent in the early stages of DKD, years before renal failure; however, miRNA profiles at this early stage have not been investigated. Long-term observations have revealed that microalbuminuria is reversed in one-third of the patients after a decade and less than half the patients develop macroalbuminuria; this is more pronounced in patients with T2DM [26]. Initially elevated miRNA levels are eventually reduced during severe renal failure. In this study, we examined patients with T2DKD (G1-2A3), paving the way for developing methods to diagnose incipient T2DKD.

UExo-derived miRNA profiles of patients with CKD showed that the expression of miR-877-3p was increased and that of miR-15a-5p was decreased [22]. In addition, the expression of miR-877-3p was markedly upregulated in interstitial pulmonary fibrosis [27], and circulating miR-15a-5p was negatively correlated with blood glucose levels [28]. Although there is no evidence for the association of these miRNAs with DKD, the findings in this study suggested that miR-877-3p and miR-15a-5p mediate fibrosis in DKD and are differentially expressed in patients with DKD and DM. These miRNAs might be useful for characterizing the similarities and differences between CKD and DKD; additional studies are needed to distinguish DKD from unexplained CKD.

The role of miRNAs in CKD progression has been studied extensively. Muralidharan et al. had found that the expression levels of 384 miRNAs from urine and 266 circulating miRNAs differed significantly between patients with CKD with eGFR < 30 mL/min/1.73 m^2^ and those with eGFR ≥ 30 mL/min/1.73 m^2^ [29]. Some miRNAs that are involved in CKD also have similar regulatory roles in DKD. In TGF-*β*1-induced NRK52E cells, the expression of let-7b is decreased, while the activity of Smad3 is enhanced [30]. These changes have been observed both in early and advanced DKD mice and in a nondiabetic renal fibrosis model [31]. One factor that promotes kidney fibrosis is miR-21, which is distributed in the cortical glomeruli and renal tubular cells; it modulates the expression of matrix metalloproteinase-9 (tissue metalloproteinase-1, MMP9/TIMP1). Additionally, miR-21 negatively regulates Smad7 to promote kidney damage [32] and plays a role in promoting fibrosis via multiple pathways in nondiabetic nephropathies such as IgA nephropathy [33, 34].

Unfortunately, patients with DKD rarely undergo renal biopsies, and so, we do not often detect differentially expressed UExo-derived miRNAs in diabetic kidneys. Previous studies have shown that glomerular changes were attenuated ten weeks after modeling, with improved albuminuria [35]. We found that the level of miR-877-3p also increased in mice renal cortex. MiR-362-3p is known to be upregulated in human kidney allografts with tubulointerstitial fibrosis [23]. MiR-150 promotes the aging of renal glomerular mesangial cells and renal fibrosis [36]. The levels of UExo-derived miRNAs may be differentially altered in kidneys, and urinary exosomes could transport miRNAs to distant target cells. These differentially expressed UExo-derived miRNAs could have two fates: they are either discharged or elevated in the kidney. Our results indicated that miR-877-3p belonged to the latter group, which is identical in kidney and urine samples.

We also compared the miRNAs identified in this experiment with those previously reported to be associated with DKD. Both miR-638 [19] and miR-133b [37] have been shown to be upregulated in urinary exosomes in patients with T2DKD. Consistent with these previous findings, we detected the differential expression of these miRNAs as well. The expression of miR-638 is known to be elevated in renal tubulointerstitial injury. In renal biopsies of patients with lupus nephritis, the expression of miR-638 was shown to be decreased in the glomeruli. However, tubulointerstitial miR-638 was increased and significantly correlated with proteinuria and the disease activity score [38]. In contrast, miR-133b expression was shown to be decreased in patients with polycystic kidney disease [39].

For the further explanation, we performed gene ontology (GO) function and KEGG pathway annotations for all ten miRNAs. For miR-877-3p, we found that the biological processes included “cell death,” “epidermal growth factor receptor signaling pathway,” and “response to hypoxia,” and the KEGG pathways enriched included “p53 signaling pathway,” “FoxO signaling pathway,” and “Fatty acid biosynthesis and metabolism.” Many studies have shown that these biological processes and pathways were closely related to DKD. Chronic hypoxia of the tubulointerstitium has been recognized as an important early event in diabetes mellitus [40]. Therefore, miR-877-3p might be involved in oxidative stress and sympathetic denervation of the kidney due to autonomic neuropathy, causing tubular apoptosis. Kato et al. had reported that Akt/FoxO pathway regulation might be a novel mechanism by which TGF beta induces unopposed MC survival and oxidant stress during early DKD [41]. These results suggested that miR-877-3p might increase FoxO3a phosphorylation and transcriptional inactivation via PI3K/Akt, thus accelerating renal disease.

The p53 (apoptosis-induced nuclear transcription factor), mammalian target of rapamycin (mTOR), and AMP-activated protein kinase (AMPK) pathways are known to play important roles in DKD. In a KEGG analysis, the predicted target genes of miRNAs were found to be related to p53, mTOR, AMPK, malignancies, neurotrophic factors, and other signaling pathways. These results suggested that these miRNAs might also promote the expression of PIGs through the p53 pathway, thus activating the ROS system. Based on the results of the KEGG pathway analyses, we speculated that the elevated expression of miRNAs such as miR-362-3p could mediate the activation of the Akt/mTOR pathway in diabetic podocytes. AMPK activation depends on the sestrin 2 protein (SESN2). Based on the predicted target genes, miR-15a-5p could target the regulation of SESN1, as SESN1/AMPK also has the potential to prevent fibrosis of diabetes mellitus function.

The miRNAs identified in our study might play important roles in the pathogenesis and progression of DKD, as evidenced by the high stability of UExo-derived miRNAs, compared to the free miRNAs in body fluids. A single miRNA can regulate multiple target genes, and multiple miRNAs can target a single gene. This was merely a cross-sectional study, and basic experimental studies and cohort studies are still needed. Recently, ultrasound-microbubble-mediated gene transfer has been applied to treat renal, peritoneal, hepatic, and other fibrosis conditions [42]. The microbubble technology has been used to knockdown miR-21 expression in db/db mice, which alleviated microalbuminuria, renal fibrosis, and inflammation [43].

## 5. Conclusions

Although single miRNAs could be used as sensitive biomarkers for the diagnosis of DKD, they might exhibit poor specificity, as many miRNAs have regulatory roles in both DKD and non-DKD. Many studies have examined UExo-derived miRNAs using the microarray approach; we identified a number of new DKD-related miRNAs such as miR-362-3p, miR-877-3p, miR-150-5p, and miR-15a-5p, which might be novel candidate biomarkers for incipient DKD, especially miR-877-3p. Using multiple miRNAs to diagnose DKD could improve the specificity. By assessing the effect of each miRNA on DKD, we can weigh the relevant miRNAs using a formula to cover all the differentially expressed miRNAs to accurately assess the risk of DKD, thus contributing to early diagnosis. In general, UExo-derived miRNAs might be suitable biomarkers for detecting DKD, as well as potential therapeutic targets.

## Supplementary Material

Table S1 RNA Quantification and Quality Assurance by NanoDrop ND−1000. Figure S1 Electropherograms image of Urinary Exosomes by Agilent 2100 Bioanalyzer. Figure S2 miRNA Target Gene Network.

## Figures and Tables

**Table 1:** Clinical and laboratory parameters of patients with type 2 diabetes.

	Normoalbuminuric DM2	Macroalbuminuric DM2
*N*	5	5
Age (years)	53.40 ± 3.90	54.60 ± 2.98
Gender (male/female)	3/2	3/2
Diabetes duration (years)	8.00 ± 1.58	9.20 ± 1.66
SBP (mmHg)	116.80 ± 4.88	118.40 ± 3.70
DBP (mmHg)	76.20 ± 3.07	76.60 ± 3.31
HbA1C (%)	8.58 ± 0.76	9.20 ± 0.92
Body mass index (kg/m^2^)	26.18 ± 1.42	25.75 ± 2.45
Serum creatinine (*μ*mol/L)	66.4 ± 9.17	66.60 ± 7.00
eGFR (mL/min × 1.73 m^2^)	99.78 ± 8.41	100.60 ± 5.38
Triglycerides (mmol/L)	2.10 ± 0.26	1.53 ± 0.27
Cholesterol (mmol/L)	5.08 ± 0.36	4.78 ± 0.42
LDL-C (mmol/L)	3.32 ± 0.29	3.09 ± 0.22
HDL-C (mmol/L)	0.92 ± 0.58	1.03 ± 0.15
Retinopathy (y/n)	2/3	5/0
ACR (mg/mmol)	0.92 ± 0.35	30.90 ± 3.92^∗^
AER (mg/24 h)	11.60 ± 2.52	402.60 ± 24.738^∗^

Data are shown as mean ± SEM; DM2: type 2 diabetic patients; SBP: systolic blood pressure; DBP: diastolic blood pressure; HbA1C: glycosylated hemoglobin; GFR: glomerular filtration rate; LDL-C: low-density lipoprotein cholesterol; HDL-C: high-density lipoprotein cholesterol; ACR: albumin/creatinine ratio; AER: albumin excretion rate; ^∗^*P* < 0.001, macroalbuminuric versus normoalbuminuric patients.

**Table 2:** Differentially expressed miRNAs.

Name	Fold change (DKD versus DM)	*P* value
hsa-miR-362-3p	21.88235	0.001172371
hsa-miR-877-3p	4.261229	9.5308*e* − 08
hsa-miR-150-5p	3.477725	0.000107498
hsa-miR-491-5p	2.29545	0.031921743
hsa-miR-133b	2.262081	0.003187535
hsa-miR-638	2.186886	5.58287*e* − 06
hsa-miR-186-5p	2.144811	0.00043897
hsa-miR-324-3p	2.061286	0.004536979
hsa-miR-15a-5p	0.373129	0.011076438
hsa-miR-3191-5p	0.003389	2.57342*e* − 07

DKD: diabetic kidney disease; DM: diabetic patients.
