# Corrigendum to “Urinary Exosomal MicroRNA Profiling in Incipient Type 2 Diabetic Kidney Disease”

**DOI:** 10.1155/2018/5969714

**Published:** 2018-02-27

**Authors:** Yijun Xie, Yijie Jia, Cuihua Xie, Fang Hu, Meng Xue, Yaoming Xue

**Affiliations:** Department of Endocrinology and Metabolism, Nanfang Hospital, Southern Medical University, Guangzhou, Guangdong 510515, China

In the article titled “Urinary Exosomal MicroRNA Profiling in Incipient Type 2 Diabetic Kidney Disease” [1], the Authors' Contributions section was missing and should be added as follows:
