# Supplementary material for: Urinary Exosomal MicroRNA Profiling in Incipient Type 2 Diabetic Kidney Disease
Source: J Diabetes Res. 2017 Sep 5;2017:6978984. doi: 10.1155/2017/6978984 (PMC5605810; doi:10.1155/2017/6978984)

Table S1 RNA Quantification and Quality Assurance by NanoDrop ND-1000

| **Sample ID** | **OD260/280 Ratio** | **OD260/230 Ratio** | **Conc. (ng/μl)** | **Volume (μl)** | **Quantity (ng)** | **QC result**  **Pass or Fail** |
| --- | --- | --- | --- | --- | --- | --- |
| DKD 1 | 1.63 | 1.41 | 50.99 | 15 | 764.85 | Pass |
| DKD 4 | 1.51 | 0.58 | 84.81 | 15 | 1272.15 | Pass |
| DKD 5 | 1.58 | 0.83 | 63.69 | 15 | 955.35 | Pass |
| DKD 6 | 1.60 | 0.89 | 60.11 | 15 | 901.65 | Pass |
| DKD 7 | 1.62 | 1.11 | 48.21 | 15 | 723.15 | Pass |
| DM6 | 1.66 | 1.28 | 65.84 | 15 | 987.60 | Pass |
| DM7 | 1.61 | 1.65 | 48.33 | 15 | 724.95 | Pass |
| DM8 | 1.55 | 1.41 | 46.26 | 15 | 693.90 | Pass |
| DM9 | 1.59 | 1.18 | 35.79 | 15 | 536.85 | Pass |
| DM10 | 1.53 | 0.56 | 67.10 | 15 | 1006.50 | Pass |

Figure S1 Electropherograms image of Urinary Exosomes by Agilent 2100 Bioanalyzer


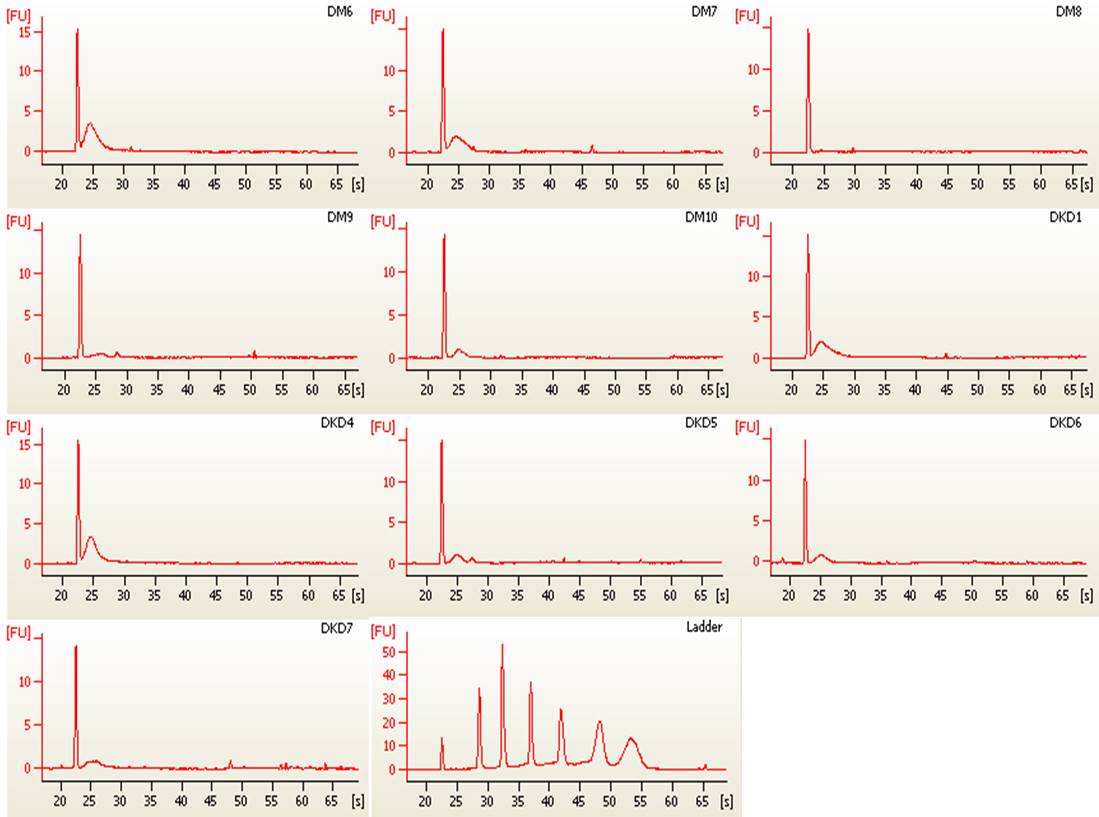


Figure S2 miRNA Target Gene Network


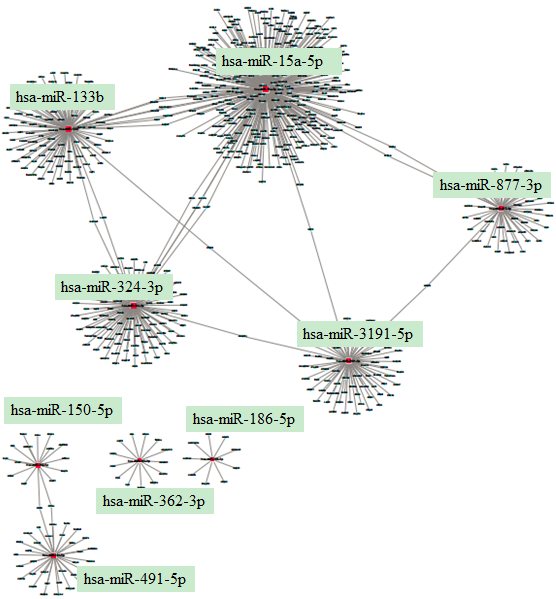

Supplement: Supplementary file 1 — Table S1 RNA Quantification and Quality Assurance by NanoDrop ND−1000. Figure S1 Electropherograms image of Urinary Exosomes by Agilent 2100 Bioanalyzer. Figure S2 miRNA Target Gene Network. [file 6978984.f1.docx]
